# Assessment of ApoC1, LuzP6, C12orf75 and OCC-1 in cystic glioblastoma using MALDI–TOF mass spectrometry, immunohistochemistry and qRT-PCR

**DOI:** 10.1007/s00795-019-00223-8

**Published:** 2019-04-20

**Authors:** Petros Evangelou, Mathias Groll, Henry Oppermann, Frank Gaunitz, Christian Eisenlöffel, Wolf Müller, Klaus Eschrich, Anne Schänzer, Ulf Nestler

**Affiliations:** 1 Department of Neurosurgery, University Hospital Leipzig, Liebigstrasse 20, 04103 Leipzig, Germany; 2 Institute of Neuropathology, University Hospital Leipzig, Liebigstrasse 26, 04103 Leipzig, Germany; 3 Rudolf Schoenheimer Institute of Biochemistry, Medical Faculty, University of Leipzig, Johannisallee 30, 04103 Leipzig, Germany; 4 Institute of Neuropathology, Justus Liebig University Giessen, 35392 Giessen, Germany

**Keywords:** ApoC1, C12orf75, Cystic glioblastoma, LuzP6, MALDI–TOF, Malignant glioma, OCC-1

## Abstract

Mass spectrometric analysis of glioblastoma cyst fluids has disclosed a protein peak with *m*/*z* 6424–6433. Among the proteins, potentially generating this peak are ApoC1 and LuzP6. To further elucidate protein expression of glioblastoma cells, we analyzed MALDI–TOF results of cyst fluid, performed immunohistochemistry and mRNA analysis. MALDI–TOF protein extraction from 24 glioblastoma cyst fluids was performed with a weak cation exchange. 50 glioblastoma samples were stained with two custom-made antibodies against LuzP6 and commercial antibodies against ApoC1, C12orf75 and OCC-1 and analyzed. For mRNA detection, 16 tissue samples were stored in RNAlater, extracted using the miRNeasy kit and reversely transcribed. For 12 patients, synopsis of results from all three examinations was possible. MALDI–TOF confirmed the peak at 6433 Da in 75% of samples. Immunohistochemically, LuzP6 was detected in 92% (LuzP6_1–29_) and 96% (LuzP6_30–58_) of samples and ApoC1 in 66%. Mean mRNA levels were highest for ApoC1, followed by LuzP6. No correlation between mRNA expression, immunohistochemical staining and intensity of the MALDI–TOF peaks was found. An unequivocal identification of one protein as the source for the 6433 peak is not possible, but our results point to ApoC1 and LuzP6 as the underlying proteins.

**Electronic supplementary material:**

The online version of this article (10.1007/s00795-019-00223-8) contains supplementary material, which is available to authorized users.

## Introduction

In a preceding study, SELDI–TOF analysis of glioblastoma cyst fluid had revealed a number of protein peaks with significantly increased occurrence, when comparing cyst content to cerebrospinal fluid (CSF) from patients with spinal stenosis. Most predominantly, a protein with *m*/*z* 6424 (6405–6443 Da) was found [1]. MALDI–TOF spectrometry confirmed this peak in a more recent set of cyst fluid samples, still with a significant difference between glioblastoma cyst fluid and CSF, but with a somewhat higher molecular weight (6433 *m*/*z*, Appendix Fig. 1).

Taking into account a technical variance of 0.3% when determining molecular weights (e.g.,  ± 19 Da), the two peaks can be considered to refer to the same glioblastoma protein. Searching the Expasy TagIdent tool for a human protein of 6424 Da revealed apolipoprotein C1 (ApoC1), leucine zipper protein 6 (LuzP6) and overexpressed in colon carcinoma 1 protein (OCC-1) as potential candidates. For a size of 6433, only ApoC1 and LuzP6 remained [2]. The experiments presented here were conceived to identify one of the candidates as the underlying protein.

Most recently, two further proteins are displayed by TagIdent at 6433 Da (accessed October 1, 2018), the spermatid nuclear transition protein 1 (chromosome 2q35) and the small integral membrane protein 27 (chromosome 9p21.1), which have not been included in the present study.

ApoC1 is a secreted, lipid transport protein, with serum concentrations around 60 µg/ml and CSF concentrations about 0.8 µg/ml [3, 4]. It is mainly synthesized in the liver and is involved in forming VLDL and HDL lipoproteins. A number of splice variants and isoforms exist, among them a protein of 6632 Da and a truncated subform of 6432 Da. The latter consists of 55 amino acids with an isoelectric point at 8.24. ApoC1 is encoded on chromosome 19q13.2, in a region that can be altered during codeletion of 1p19q in oligodendrogliomas or astrocytomas [5]. ApoC1 protein has been colocalized with GFAP in human hippocampal cells, and cultured astrocytes can express ApoC1 mRNA [6]. The ApoC1 gene is expressed during differentiation of peripheral blood monocytes to macrophages and silencing of the gene leads to decreased LDL uptake in macrophages [7, 8]. A few reports have discussed the presence of ApoC1 protein in non-CNS tumors, among them, pleural mesothelioma, gastric adenocarcinoma or prostate cancer [9].

The leucine zipper protein 6 (LuzP6, or myeloproliferative disease-associated 6 kDa antigen MPD6) is a cryptic, putative tumor-self antigen, associated with myeloproliferative disease [10]. It has a molecular weight of 6437 Da and consists of 58 amino acids (p*I* 9.69). The gene is located on chromosome 7q33 and an induction of the protein by interferon alpha treatment has been discussed [10]. Translation starts from an atypical AUU codon in the 3′ terminal region of the myotrophin gene.

The overexpressed in colon carcinoma 1 protein (OCC-1 or adipogenesis down-regulated 3 AGD3) has a molecular weight of 6407 Da, 63 amino acids and an isoelectric point of 6.11. It is encoded on chromosome 12q23.3, in open reading frame 75 (C12orf75). First results had suggested a non-coding regulatory effect of the OCC-1 mRNA in cancer tissue. Further research additionally showed expression and down-regulation of the protein during differentiation of mesenchymal stem cells to adipocytes [11, 12].

The aim of the study was to determine by immunohistochemistry and qRT-PCR, which of the four proteins constitutes the source of the mass spectrometric peak at 6433 Da.

## Materials and methods

A positive vote from the ethic committee of the Faculty of Medicine at the University of Leipzig had been obtained prior to the collection of specimens and analysis of patient data (#330-13-18112013). The patients provided written informed consent, and an overview concerning the demographic data is given in Table 1.

**Table 1:** Patient demographics

	MALDI–TOF	Immunohistochemistry	qRT-PCR	Synopsis
Samples	24	50	16	12
Patients	21	47	15	12
Male	76.2%	63.8%	60.0%	66.7%
Mean age (years)	59.2	57.6	64.7	65.3
Cystic tumors	100%	86%	94%	100%

### MALDI–TOF mass spectrometry

In total, 24 glioblastoma cyst fluids from 21 patients were examined and the results compared to 23 CSF samples from non-tumor patients [2]. Intraoperatively, the cyst was punctured before starting tumor dissection and the aspirated fluid was cooled down to − 20 °C. After thawing for the first time, samples were centrifuged at 1000×*g* for 5 min. The supernatant was distributed into aliquots and frozen at − 20 °C until use. CSF samples were treated accordingly.

After thawing an aliquot, tumor cyst fluid supernatant was diluted 1:5 in 0.5 M TRIS buffer pH 6.8, and the CSF supernatant was used without dilution. 30 µl of these samples was mixed with 60 µl binding buffer and 10 µl magnetic bead suspension for protein preparation by weak cation exchange (WCX kit, Bruker Daltonics). The subsequent washing steps were performed according to the instructions of the manufacturer. Finally, 1 µl of the cleaned specimen was spotted on a ground steel target, air dried and covered with 1 µl of cinnamic acid matrix (4 mg/ml HCCA in a 1:1 mixture of ACN with 0.1% TFA).

Mass spectra were recorded by MALDI–TOF MS from 1500 to 16600 mass over charge ratio (*m*/*z*) using an Autoflex II spectrometer (Bruker Daltonics) in linear positive mode with 500 shots accumulated per spectrum. The raw spectra were processed by baseline subtraction and a slight smoothing with Flex analysis 2.4 (Bruker Daltonics). From each spectrum, peaks were extracted manually, and the thresholds for peak assignment were a signal to noise ratio of at least 6 and a peak width (in half height of maximum) of 0.1 *m*/*z*. The different peak intensities in one sample were compared to that of the highest peak and expressed as relative intensities, ranging from 0 to 1.

For determining the underlying proteins related to the identified peaks, an Expasy TagIdent search was performed. The *m*/*z* ratio was entered as the molecular weight, allowing an Mw range of ± 0.3% and setting the organism to *Homo sapiens* (9606). For two peaks, no human protein was displayed with these settings, so the search was repeated with the same weight and range settings, but without restricting the species (Table 3).

### Immunohistochemistry

For the immunohistologic staining of all antigens, rabbit polyclonal antibodies were employed. In total, paraffin sections from 43 cystic glioblastomas and 7 non-cystic glioblastomas were stained for ApoC1, LuzP6, C12orf75 and Occ-1. Tissue specimens were obtained during neurosurgery as part of the routine histology. The tissue samples were processed according to standard procedures with fixation in 4% formalin followed by paraffin embedding.

The paraffin slides were processed and stained semi-automatically in a Benchmark Ultra 2 (Ventana, USA). Slices for ApoC1 staining and C12orf75 detection were preconditioned in CC1 buffer during 36 min, apart from this standard reaction buffer (pH 7.4–7.8) was used at 37 °C. The antibodies were diluted in antibody diluent (Ventana). ApoC1 and both LuzP6 antibodies were diluted 1:50 and incubated for 60 min, C12orf75 was used in a dilution 1:20 for 32 min and Occ-1 at 1:400 for 32 min. Secondary antibodies (ultraView Universal HRP multimer, Ventana) were visualized semi-automatically with the ultraView Universal DAB detection kit or AEC staining, followed by hematoxylin/bluing counterstaining.

In anti-ApoC1 (HPA051518, Sigma-Aldrich), the immunogen consisted of the amino acids 28–77 of the protein (28 PDVSSALDKL KEFGNTLEDK ARELISRIKQ SELSAKMREW FSETFQKVKE 77), mainly corresponding to the processed truncated apolipoprotein C1 (29–83, 6432 Da). Liver tissue served as positive control with staining of cytoplasmic droplets in the vicinity of the endoplasmic reticulum. Positive staining is regularly found in endothelial cells, and can be detected in astrocytes.

For anti-LuzP6, two custom-made antibodies were manufactured (Abnova, Taiwan) according to the commanded design (UN). The peptides were synthesized by the manufacturer and conjugated to KLH before immunization of the animals. The first immunogen consisted of the 29 n-terminal amino acids LuzP6_1–29_(1 MKSVISYALY QVQTGSLPVY SSVLTKSPL 29) and the second immunogen of the 29 c-terminal amino acids LuzP6_30–58_ (30 QLQTVIYRLI VQIQHLNIPS SSSTHSSPF 58) of the LuzP6 protein.

**Fig. 1:**
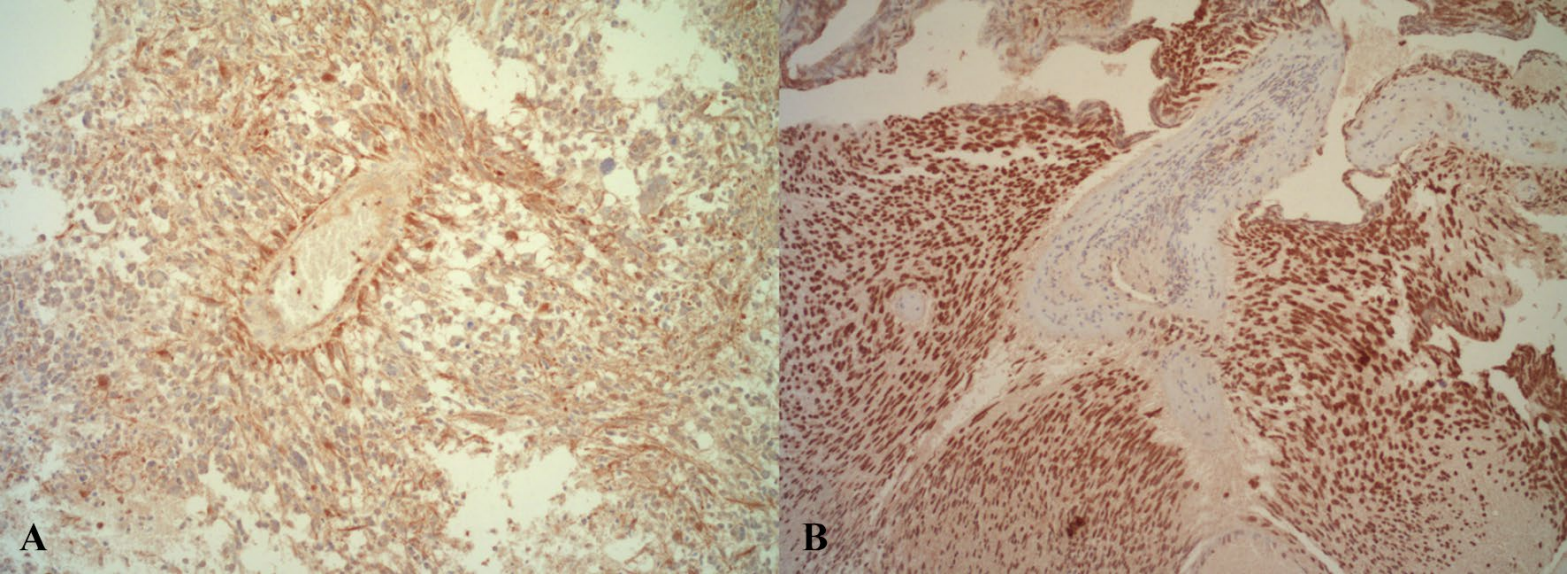
Strong positive LuzP6_30-58_ immunohistochemical staining of cystic glioblastomas. **a** Light brown cytoplasmatic staining of astrocyte-shaped cells around a vessel. **b** Dark brown nuclear staining (both original magnification 200×)

At first, biopsies of Burkitt lymphoma tissue were employed as positive control, but during establishing the staining procedure it became obvious that the striated ducts of salivary glands show a strong positivity (Appendix Fig. 2). In glioblastoma tissue, two typical staining patterns were observed: first, a weak cytoplasmatic staining of astrocyte-shaped cells; and second, a strong nuclear stain (Fig. 1). In general, comparing the two antibodies, the C-terminal antibody LuzP6_30–58_ revealed a more intense and distinct coloring of the cells than the N-terminal antibody LuzP6_1–29_. In addition, LuzP6_30–58_ displayed unique staining of the lamina elastica interna in muscular arteries (Appendix Fig. 3).

### C12orf75

The anti-C12orf75 antibody (#HPA 065311, Sigma Aldrich) was generated from a 62 amino acid recombinant fragment of human C12orf75 (1 MGCGNSTATS AGAGQGPAGA AKDVTEESVT EDDKRRNYGG VYVGLPSEAV NMVSSQTKTV RK 62). As positive control, kidney tissue was employed. According to The Human Protein Atlas, positive staining is generally present in neuropil structures, but also in cortical pyramidal cells (https://www.proteinatlas.org).

For the anti-Occ1 antibody (#PAB16693, Abnova, recently re-named “C12orf75 polyclonal antibody” by the manufacturer), a synthetic protein including 15 n-terminal amino acids of human C12orf75 was used as immunogen (22 NVHRHFQGNS KQSPF 36). During further molecular workup, this turned out to correlate to an erroneous translation, so that the anti-Occ1 antibody staining was established to serve as negative control for the four other antibodies [12]. Spleen tissue was used to establish the staining protocol and peripheral blood granulocytes were found to display a strong positive signal.

Slides were assessed independently by two examiners (PE, UN) using a semiquantitative scale (Table 2). The staining intensity of glioblastoma cells was ranked from 0 (none) to 3 (dark) and the percentage of fields of view with stained glioblastoma cells in relation to all fields of view in the slide was determined. Both values were multiplied and the result was averaged between examiners.

**Table 2:** Assessment scale for immunohistologic staining

Intensity		Number		Intensity × number	
0	No staining	0	0%	0, 1, 2	Negative
1	Weak	1	Less than 10%	3, 4	Weak
2	Medium	2	10–50%	6, 8	Medium
3	Strong	3	51–80%	9, 12	Strong
		4	More than 80%		

Statistical analyses were performed with SPSS software and GraphPad QuickCalcs using the test methods as detailed in the “Results” section.

### qRT-PCR

From 15 glioblastoma patients, 16 samples were processed for mRNA detection. After neurosurgical dissection, tissue samples were immediately transferred into RNAlater solution (Qiagen, Hilden, Germany) and stored at 4 °C. 40–80 mg of the tissue was employed for RNA extraction using the miRNeasy kit (Qiagen) without DNase digest and RNA samples were stored at − 80 °C. Random primer PROMEGA Improm II kit was used for reverse transcription into cDNA, which was then stored at − 20 °C until further use. Quantitative PCR was performed with Thermo Scientific SYBR green in triplicate using the primer pairs disclosed below. Quantification of RNA was done in a Rotor-Gene Q (Qiagen) and the resulting photometric counts were averaged and normalized against mRNA encoding TATA box binding protein (TBP) as reference gene. Results were additionally plotted in comparison to a commercial reference sample of human brain occipital lobe cDNA (BioCat, Heidelberg, Germany).

The following primer pairs were chosen:

ApoC1_172: 5′-CCA GAC GTC TCC AGT GCC TTG GAT AA-3′;
ApoC1_321: 5′-CTC CTT CAC TTT CTG AAA TGT CTC TG-3′;
C12orf75_316: 5′- AGC CAA AGA TGT AAC AGA AGA ATC CG -3′;
C12orf75_405: 5′-ACA GCT TCA GAT GGT AGG CCA AC-3′;
LuzP6_3197: 5′-AGT CAT TTC ATA TGC ACT ATA TCA AG-3′;
LuzP6_3353: 5′-GCT ATG CGT AGA AGA ACT ACT T-3′;
Occ1_465: 5′-GAC TCA AGA ATC AAG AGC TTG CTC AT-3′;
Occ1_644: 5′-TTC CCT TCT ATT ACC ACA ACA ATA TG-3′.

The primers were designed to include a sequence of about 100–200 base pairs and were checked for self-assembling. In the LuzP6 gene there are no introns, whereas in the three other gene sequences the primers cover more than one exon of the mRNA.

## Results

### MALDI–TOF mass spectrometry

The MALDI–TOF assessment revealed 148 peaks in the 24 samples. They were sorted according to their mean relative intensity and the percentage of positive samples was noted, together with potential protein candidates (Table 3). The protein candidates for 6433 and 6632 Da both comprised ApoC1, and for 8564 Da ApoA2. Searching the peaks 1865 and 1778 did not result in known human proteins, but for both sizes HIV gag polyprotein was returned.

**Table 3:** Size of the ten protein peaks with the highest mean relative intensities from MALDI–TOF analysis of glioblastoma cyst fluid samples (*n* = 24)

Dalton	Relative intensity	% Positive samples	Difference to CSF (*p* value)	Potential underlying proteins (Expasy TagIdent^a^)
6433	0.36	75.0	0.0004	ApoC1, LuzP6, Sim27, Stp1
1865	0.23	29.2	0.0094	No human proteins: HIV-1 Gag polyprotein spacer peptide 2
4967	0.20	54.2	ns	Ca2d4, Cstf3, Hmhb1, Tjap1
7565	0.18	33.3	ns	Ccl18, Hcst, Rad1, Sg1d1
6632	0.17	62.5	0.0189	ApoC1, cholecystokinin, Cxl17, RS30
2661	0.16	29.2	0.0479	Humanin-like 5, humanin-like 8
8564	0.12	66.7	ns	ApoA2, Hsbp1, interleukin-8, ubiquitin
3436	0.09	25.0	ns	Histatin-1, insulin B-chain
3851	0.09	25.0	ns	Tumor necrosis factor intracellular domain 2, insulin-like 3B chain
1778	0.09	20.8	0.0496	No human proteins: HIV-2 Gag polyprotein spacer peptide 1, mammalian neurogranins

*p* value obtained by two-sided Fisher’s exact test using a 2 × 2 contingency table

*ns* not significant

^a^Expasy TagIdent accessed October 13, 2018: Mw as given, Mw range 0.3%, organism 9606 or none

**Fig. 2:**
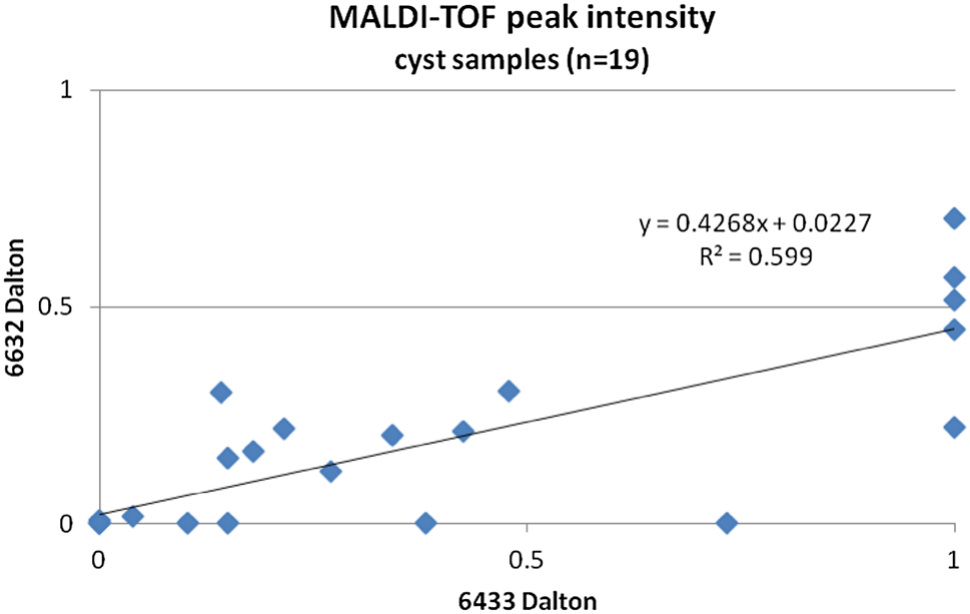
Significant correlation between relative peak intensities in the 19 samples containing peaks at 6433 and 6632 Da. Both protein bands can result from ApoC1

Four MALDI–TOF peaks were found in more than half of the samples, namely at 6433, 8564, 6632 and 4967 Da. Their relationship concerning the mean relative intensities was examined by linear regression analysis and showed a significant correlation between the peaks at 6433 Da and 6632 Da (Fig. 2). The further peaks did not correlate with each other.

### Immunohistochemistry

Glioblastoma samples were positive for LuzP6_1–29_ in 92% and for LuzP6_29–50_ in 96%. Positive ApoC1 staining was found in 66%, C12orf75 in 48% and Occ1 in 14%. The number of samples with strong positivity was significantly higher for the antibodies LuzP6_1–29_ and LuzP6_30–58_ compared to the other groups, whereas for ApoC1, C12orf75 and Occ1 it was significantly lower (Table 4).

**Table 4:** Percentage of negative and strongly positive samples, compared to the mean values of all five antibody stainings (*n* = 50)

Percent of samples						
Staining score	ApoC1	LuzP6_1-29_	LuzP6_30-58_	Occ1	C12orf75	Mean
Negative	34.0	8.0**	4.0**	86.0**	52.0*	36.8
IRS > = 9	6.0*	40.0**	44.0**	0.0**	4.0**	18.8

Chi square test for difference to mean percentage of all five stainings: *p < 0.05 **p < 0.01

**Fig. 3:**
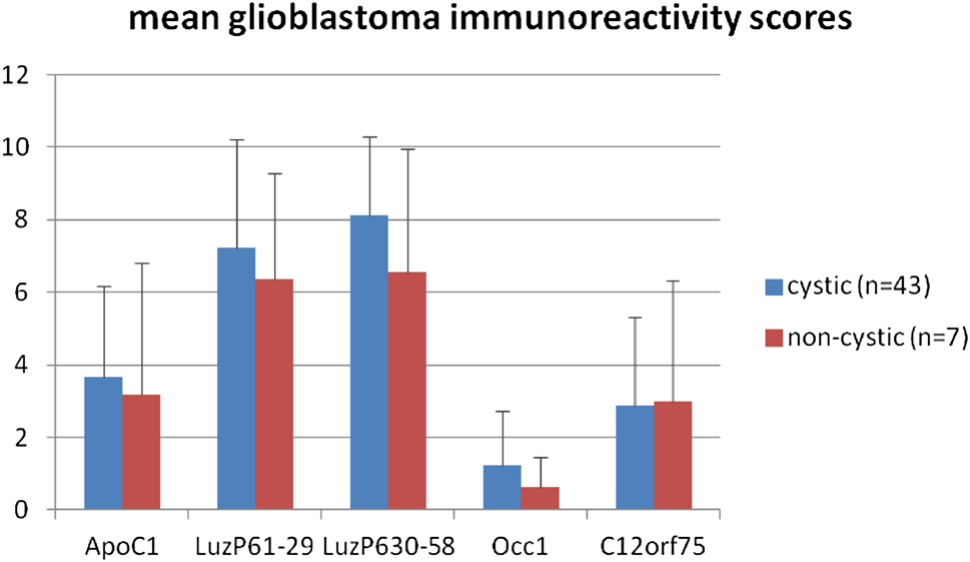
Mean values of the semiquantitative immunoreactivity score for the five antibodies, separately shown for cystic and non-cystic glioblastomas. Bars show standard deviation

The highest positive immunoreactivity score values were found for LuzP6, followed by ApoC1, C12orf75 and Occ1 (Fig. 3).

### qRT-PCR analysis

**Fig. 4:**
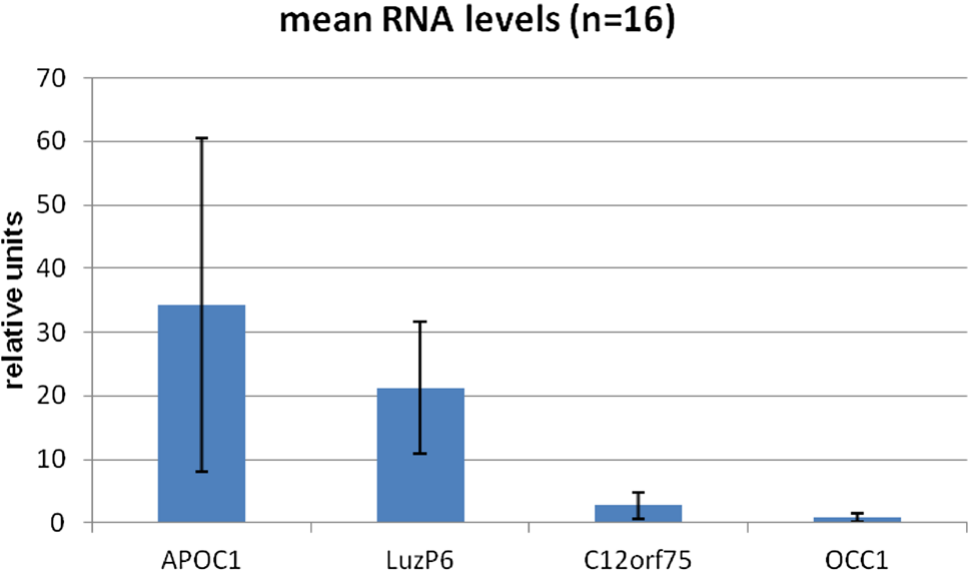
Mean RNA levels of the four proteins in 16 glioblastoma samples. Bars give the standard deviation

**Fig. 5:**
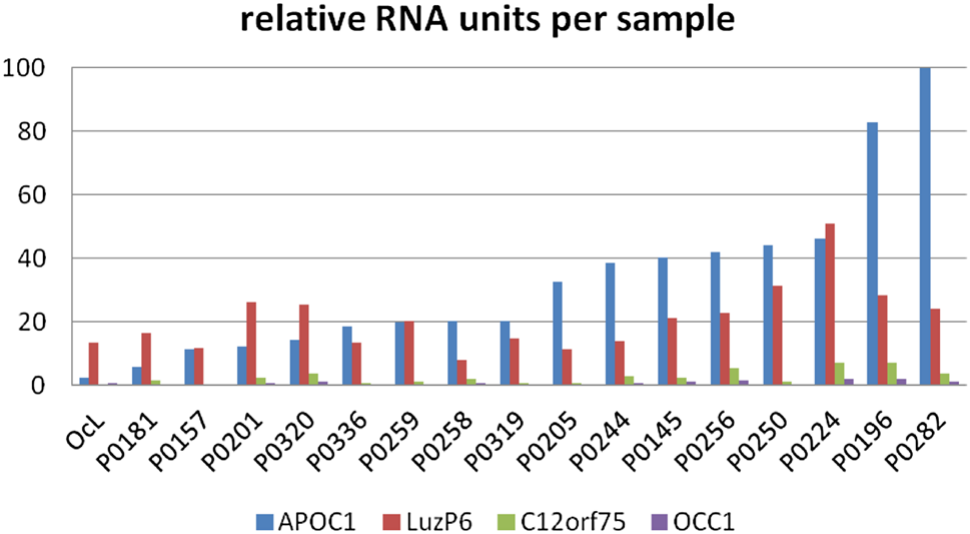
Results of RNA detection in the 16 samples and the OcL control

qRT-PCR analysis was performed using the cycles-to-threshold method, resulting in relative units, normalized to the reference mRNA. Among the 16 samples, the highest values were found for ApoC1, followed by LuzP6, C12orf75 and Occ1 (Fig. 4). For all four primer pairs, cDNA could be detected. The mRNA levels of the reference sample OcL were among the lowest, but a clear signal for LuzP6 mRNA was obtained (Fig. 5).

While for ApoC1, C12orf75 and Occ1 exon-spanning primers were available, resulting in cDNA-transcripts from mRNA, the LuzP6 gene is transcribed without introns, so the cDNA of the 16 neurosurgically obtained samples cannot unequivocally be assigned to messenger RNA.

### Comparison

For 12 samples, synopsis of triple assessment was possible. In these patients, harvesting of cyst fluid for MALDI–TOF, tissue for RNA isolation and formalin-fixed tissue for immunohistochemistry were achieved at the same time during one neurosurgical procedure. Comparison of the three concurrent results was performed by linear regression and by determining Sperman’s rho.

**Fig. 6:**
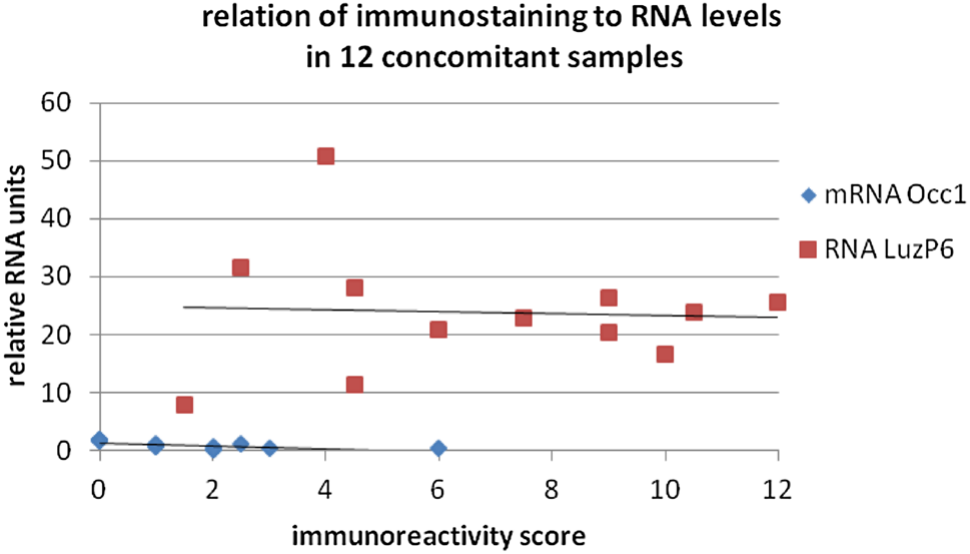
Correlation of RNA levels and immunostaining scores for Occ1 and LuzP6 in 12 concomitant samples. Nb: the staining scores for LuzP6_1-29_ and LuzP6_30-58_ were averaged. Due to the low values of Occ1 mRNA, not all 12 markers are visible in the plot

There were no significant relations, except for a significant negative correlation between Occ1 mRNA and Occ1 immunohistologic staining (Fig. 6). Given the low to negative values for both Occ1 detection techniques, and in view of its role as negative control, the result is hardly to be generalized, while a trend to lower immunostaining scores with increasing amounts of RNA in the samples was also seen for other proteins.

In four cyst fluids of the 12 patients with concurrent sampling, the MALDI–TOF peak at 6433 Da had not been detected (Table 5). The mean immunostaining scores and the mean RNA levels did not show significant differences between MALDI-positive and MALDI-negative patients when assessed by unpaired *t* test.

**Table 5:** Mean staining scores and RNA levels comparing patients without the 6433 Da MALDI–TOF peak (*n* = 4) to patients with detected peak in glioblastoma cyst fluid (*n* = 8)

	Immunostaining score					qRT-PCR units			
	ApoC1	LuzP6_1-29_	LuzP6_30-58_	Occ1	C12orf75	ApoC1	LuzP6	Occ1	C12orf75
MALDI peak 6433									
Negative	6.8	6.0	7.5	0.8	5.0	44.2	24.6	1.3	4.4
Positive	5.4	5.8	7.8	2.1	5.6	35.5	23.5	0.7	2.6

## Discussion

In the present study, the expression of the four proteins, ApoC1, LuzP6, Occ1 and C12orf75, in cystic glioblastoma is examined by three different techniques, namely MALDI–TOF mass spectrometry, immunohistochemistry and qRT-PCR. To the best of our knowledge, this study is the first report to show the cellular expression of ApoC1 and LuzP6 in glioblastoma tissue by semiquantitative comparison, and to confirm the presence of the corresponding RNA in samples of the same tumor. From the results, a pathophysiologic hypothesis on glioblastoma cyst formation can be deduced.

MALDI–TOF mass spectrometry discloses a protein band at 6433 *m*/*z* ratio, potentially corresponding to ApoC1 (6432 Da) or LuzP6 (6437 Da). The presence of a second strong peak at 6632 *m*/*z* ratio, exactly at the molecular weight of an ApoC1 isoform, puts ApoC1 in the forefront for generating these two peaks (Fig. 2, Table 3). The molecular size of C12orf75 (Occ1, 6407 Da) lies outside the ± 0.3% deviation range of the MALDI technique, lowering the probability of Occ1 contributing to the peak.

These results are supported by the RNA analyses using qRT-PCR. The highest levels are found for ApoC1 mRNA, followed by LuzP6 RNA. Messenger RNA for C12orf75 and Occ1 is detectable, but confined to the lowest levels.

In contrast to this, immunohistochemical staining reveals an abundant expression of the LuzP6 protein in glioblastoma cells, significantly higher than the expression of the three other proteins (Table 4, Fig. 3). Accordingly, the negative control, Occ1, shows significantly low expression when compared to all other samples. ApoC1 positive cells are detected, but with lower immunoreactive score levels than for LuzP6.

### ApoC1

The study demonstrates the expression of ApoC1 in glioblastoma cells, with detection of mRNA in tissue, positive immunostaining in slides and two protein peaks corresponding to the sizes of ApoC1 splice variants in glioblastoma cyst fluid. This is in concordance with literature reports that describe the protein in astrocytes and associated to brain endothelial cells [6, 13]. In the present study, ApoC1 often was detected surrounding microhemorrhage and necrotic areas (Appendix Fig. 4).

### Hypothesis on glioblastoma cyst formation

The findings of the present study suggest that ApoC1 plays a role in glioblastoma cyst formation, connected to its function in lipid metabolism and chylomicron remnant clearance. In addition, immunomodulatory functions of ApoC1, e.g., on astrocytes or microglia, have been described, though it remains under debate whether immunoprotective or immunosuppressive effects prevail [3, 4]. In general, it is accepted that ApoC1 mRNA levels increase during differentiation from peripheral blood monocytes to macrophages [8]. Most recently, ApoC1 gene silencing using siRNA was shown to result in decreased LDL uptake by monocyte-derived macrophages, indicating that ApoC1 supports phagocytic activity [7].

Hypothetically, microhemorrhage into tumor areas can lead to formation of confluent necroses. These will contain ApoC1-positive endothelial cells and induce an immunoresponse, either recruiting macrophages from peripheral blood monocytes or from activated microglia. In recruited monocytes, ApoC1 will be expressed in the developing phagocytic cells, and in microglia differentiating to phagocytic microglia, a similar ApoC1 expression seems reasonable and is supported especially by the immmunostaining data (Appendix Fig. 4).

During the immunoreactive process, foam cells and gitter cells may appear. Through fusion and continued degradation of debris, tumor cysts will arise in certain parts of glioblastoma tissue. This phagocytic cascade can also explain the presence of further protein bands in the cyst fluid, detectable by the mass spectrometric analysis, and the presence of growth factors or nutrient-like metabolites, formerly contained in the glioblastoma cells [1, 14]. Concerning the occurrence of granulocyte-bound Occ1 in the present study, the direct engulfment of polymorphonuclear neutrophils by microglia is apt to deliver Occ1 into the cyst fluid [15].

This hypothesis attributes the synthesis of the detected cyst proteins to glioblastoma cells and surrounding tissue, with a rather passive influx into the cyst fluid, without causal connection to the occurrence of cysts. The current results support this theory, since the staining intensities do not differ between cystic and non-cystic tumors (Fig. 3). Moreover, when the protein peak at 6433 Da is not present in the cyst fluid, neither a difference of immunostaining nor of RNA expression was found compared to tumors displaying the peak in the cyst (Table 5).

### Control proteins

The results presented here for the protein C12orf75 are in good concordance with data from the Human Protein Atlas (antibody HPA065311), where low to moderate immunostaining in neuropil and neuronal cells is described, as well as low RNA expression. In this study, in areas with maintained cortical structure, C12orf75 was found staining the perikarya of pyramidal cells, and in tumor areas the protein often disclosed enlarged or giant cells (Appendix Fig. 5).

The Occ1-positive cells (antibody PAB16693) comprised reproducibly a subset of polymorphonuclear granulocytes (Appendix Fig. 5). Positive tumor cells were rare and mostly displayed features of enlarged cells or even foam cells, pointing to phagocytic cells having fused with granulocytes. The hypothetical protein Occ1 has a molecular weight of 9127 Da, a peak that was not found in glioblastoma cyst fluid using MALDI–TOF MS. Nevertheless, low levels of Occ1 mRNA were detected by qRT-PCR. The small amounts correspond to the fact that only a small part of the tissue slides consists of granulocytes, potentially synthesizing the hypothetical protein. Overall, from the current results, expression of Occ1 by glioblastoma cells cannot be postulated.

### LuzP6

A most important finding is the strong and abundant positive immunostaining of glioblastoma cells by the two LuzP6 antibodies that provides evidence of LuzP6 at protein level and localizes the protein in glioblastoma tissue (Fig. 1). The results were reproducible and the staining intensities for both epitopes correlated significantly.

However, the RNA assessment is flawed due to the preparation without DNase digest and the lack of introns in the messenger RNA. This inconsistency has to be investigated in more detail, since LuzP6 is coded for on chromosome 7, which can show gains in glioblastoma and carries coding regions for EGFR and PDGFA [16].

Consequently, the next steps are immunohistochemical staining of tissue samples from further organs and tumors, especially low-grade gliomas, and detection of myotrophin by immunostaining and RNA analysis to assess whether LuzP6 and myotrophin are translated together, or even expressed as one fusion protein. Furthermore, the function of the leucine zipper region in glioblastomagenesis has to be examined, together with a potential induction of LuzP6 by glucocorticoid treatment [17].

A limitation to the conclusions of this study is the missing correlation between the 12 triple samples taken concomitantly during one neurosurgical intervention. A correlation would have enabled or reinforced the identification of a single protein as the source of the peak at 6433 Da. On one hand, though the samples are taken at the same time, they represent very different sublocations and tissue compartments of the tumor, especially in glioblastoma multiforme. On the other hand, it is known that neither DNA sequences are linearly transcribed into mRNA, nor that the mRNA is translated into protein in a certain proportion to its intracellular amount. Third, the assessment scale for the immunostaining is semiquantitative and for MALDI–TOF and qRT-PCR relative units are employed. Thus, mathematical and statistical considerations are to be interpreted cautiously.

## Conclusion

The present study shows the presence of ApoC1 and LuzP6 in glioblastoma cells. Although a final decision whether ApoC1 or LuzP6—or even both—generate the mass spectrometric peak at 6433 Da cannot be drawn from the results, the well-described functions of ApoC1 in lipoprotein metabolism and differentiation of macrophages, as well as the abundant positive immunostaining of LuzP6, warrant further research concerning glioblastoma cyst formation and glioblastoma pathogenesis in view of potential glioblastoma biomarkers.

## Electronic supplementary material

Below is the link to the electronic supplementary material.

Supplementary material 1 (DOCX 7607 kb)

## Abbreviations

ACN: Acetonitrile
ApoA2: Apolipoprotein A2
ApoC1: Apolipoprotein C1
Ca2d4: Isoform 7 of voltage-dependent calcium channel subunit alpha-2/delta-4
Ccl18: C–C motif chemokine 18
CSF: Cerebrospinal fluid
Cstf3: Cleavage stimulation factor subunit 3
Cxl17: C–X–C motif chemokine 17
EGFR: Epidermal growth factor receptor
HCCA: α-cyano-4-hydroxycinnamic acid
Hcst: Hematopoietic cell signal transducer
Hmhb1: Minor histocompatibility protein HB-1
Hsbp1: Heat shock factor-binding protein 1
KLH: Keyhole limpet hemocyanin
LuzP6: Leucine zipper protein
OcL: MRNA reference sample from human occipital lobe
PDGFA: Platelet-derived growth factor A
Rad1: Cell cycle checkpoint protein Rad1
Rs30: 40s ribosomal protein S30
Sg1d1: Secretoglobin family 1D member 1
Sim27: Small integral membrane protein 27
Stp1: Spermatid nuclear transition protein 1
TFA: Trifluoroacetic acid
Tjap1: Isoform 3 of tight junction-associated protein 1
