# Supplementary material for: Assessment of ApoC1, LuzP6, C12orf75 and OCC-1 in cystic glioblastoma using MALDI–TOF mass spectrometry, immunohistochemistry and qRT-PCR
Source: Med Mol Morphol. 2019 Apr 20;52(4):217–25. doi: 10.1007/s00795-019-00223-8 (PMC6885021; doi:10.1007/s00795-019-00223-8)

APPENDIX

Supplemental figures 1, 2, 3, 4 and 5

**Fig. 1** Visualization of protein peaks with significantly different occurrence between glioblastoma cyst fluid and CSF as detected by SELDI-TOF [1] or MALDI-TOF [2]. Depicted are the peaks with molecular weight up to 15000 Dalton, the ordinate shows 1-p, with p as statistical level of significance. Green triangles show peaks initially described by SELDI and confirmed by MALDI in the same kind of fluid: Above glioblastoma peaks at 6433 and 6632, below the CSF peak at 13871.


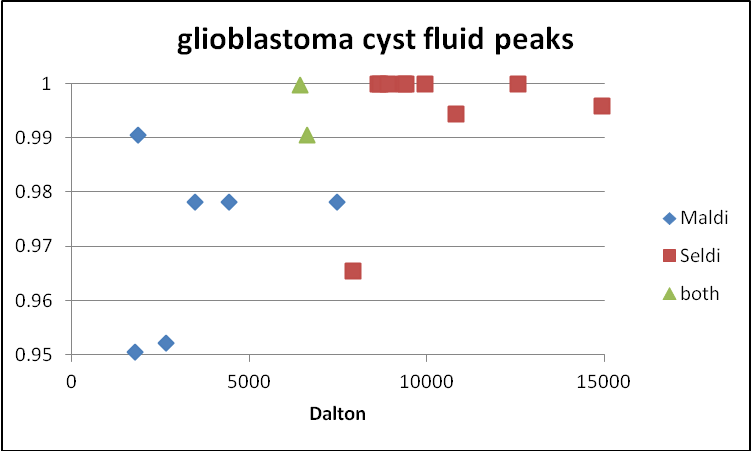

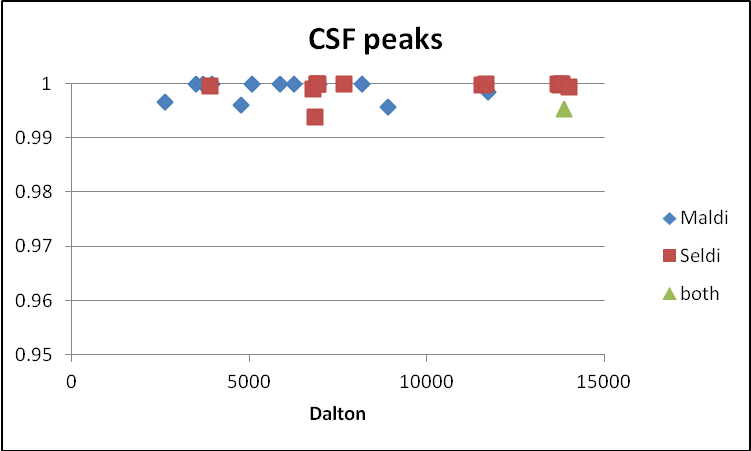


**Fig. 2** Salivary gland immunohistochemical staining for LuzP6. (A) without primary antibody, (B) LuzP6_1-29_ as primary antibody. Dark brown positive staining, especially confined to the striated ducts (original magnification 200x)


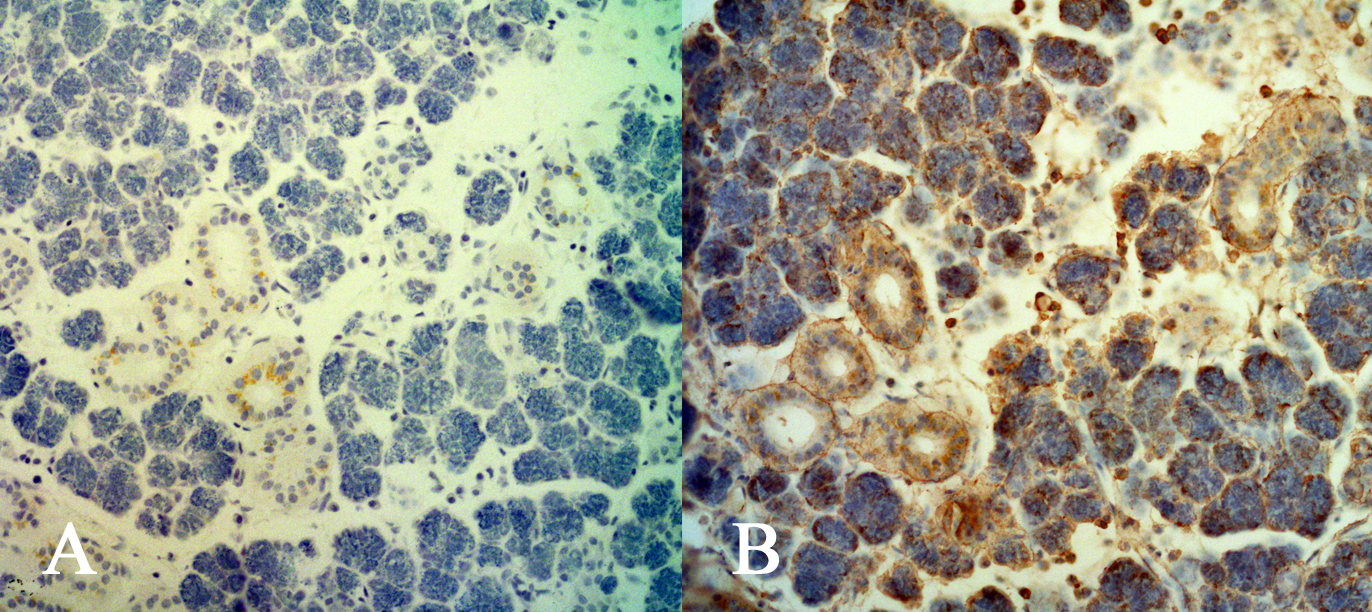
**Fig. 3** LuzP6 staining of a small muscular artery. (A) LuzP6_1-29_ (N-terminal) and (B) LuzP6_30-58_ (C-terminal), original magnification 200x. The C-terminal antibody LuzP6_30-58_(B) shows more distinct contrast between positive and negative cells, as well as characteristic staining of the lamina elastica interna


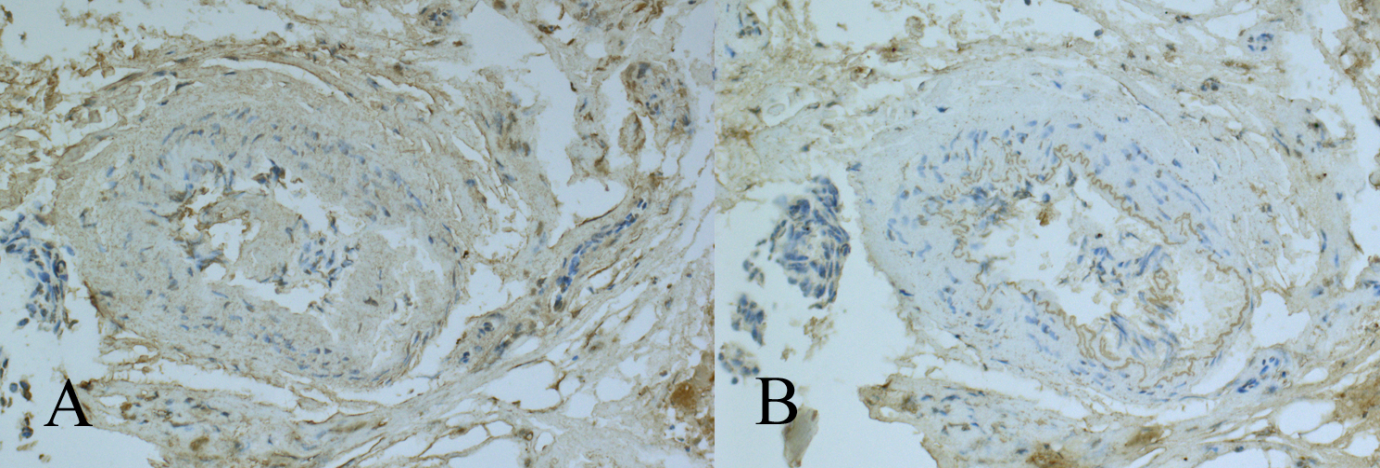

**Fig. 4** Anaplastic astrocytoma. ApoC1 immunostaining with red AEC substrate (A) Areas of hemorrhage, surrounded by ApoC1-positive cells. (B) Necrosis with surrounding ApoC1-positive staining (both original magnification 100x)


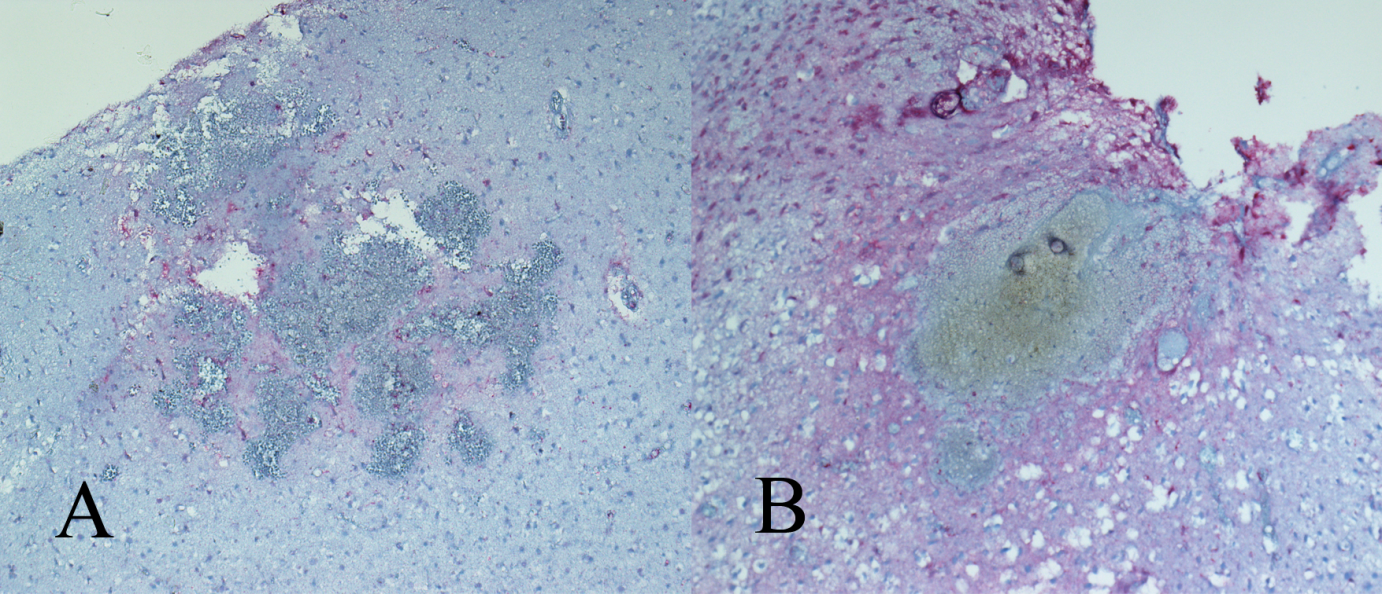


**Fig. 5** (A) C12orf75 immunostaining of glioblastoma discloses a multinucleated giant cell, together with medium cytoplasmatic staining of surrounding cells. (B) Occ1 staining of glioblastoma reveals intravascular positive polymorphonuclear cells (both original magnification 400x)


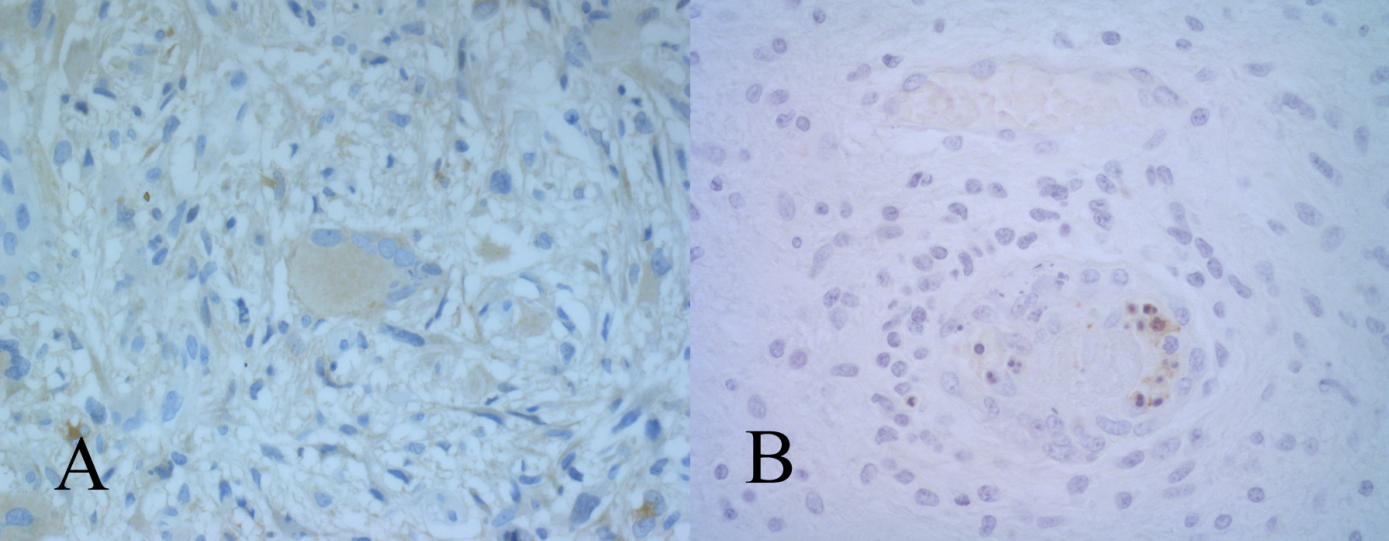

Supplement: Supplementary file 1 — Supplementary material 1 (DOCX 7607 kb) [file 795_2019_223_MOESM1_ESM.docx]
